# Complete cervical agenesis: successful surgical treatment: one case report

**DOI:** 10.11604/pamj.2020.36.211.24408

**Published:** 2020-07-23

**Authors:** Soumaya Kraiem, Olfa Zoukar, Asma Hnayin, Ahmed Zouari, Raja Faleh, Anis Haddad

**Affiliations:** 1 Department of Gynecology Obstetric, Maternity and Neonatology Center of Monastir, University of Monastir, Monastir, Tunisia

**Keywords:** Amenorrhea, congenital cervical agenesis, reconstructive surgical procedure

## Abstract

Cervical agenesis is a rare congenital pathology linked to an abnormality in the development of the Mullerian system, the mechanism of this anomaly is unknown. We reported a case of complete cervical agenesis in a 17-year-old girl who underwent a successfully utero-vaginal anastomosis.

## Introduction

Cervical agenesis is a rare Mullerian anomaly with the incidence of 1 in 80,000 to 1,00,000 live births [1]. This is a situation in which the cervix is absent and the isthmus (the lower segment of the uterus) narrows and closes, ending in the peritoneal sleeve above and away from the vaginal apex. Patients affected by this rare abnormality have a functional uterus, but without their cervix they get primary amenorrhea with cyclic pelvic pain du to hematometra. The challenge for the clinician is to restore normal periods and potentially preserve fertility.

## Patient and observation

**Figure 1:**
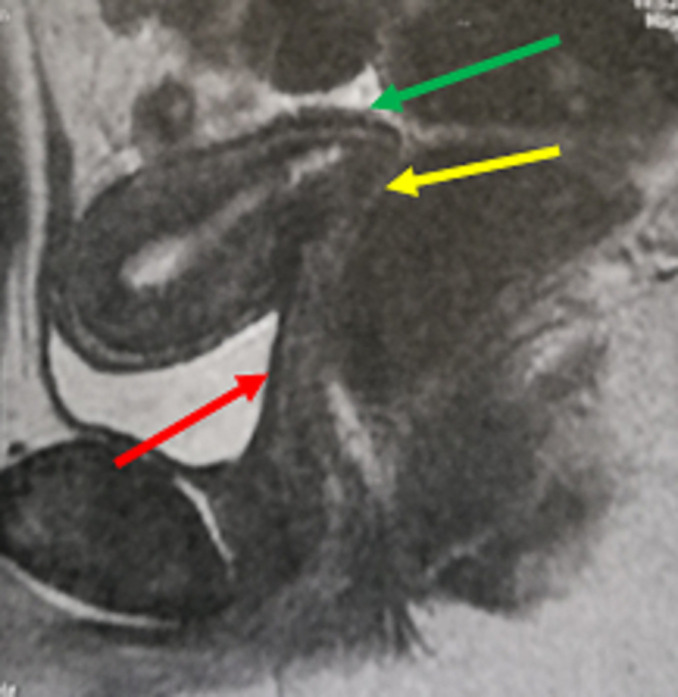
magnetic resonance imaging scan showing the vagina (in red), the isthmus (in green) and the absence of cervix (in yellow)

**Figure 2:**
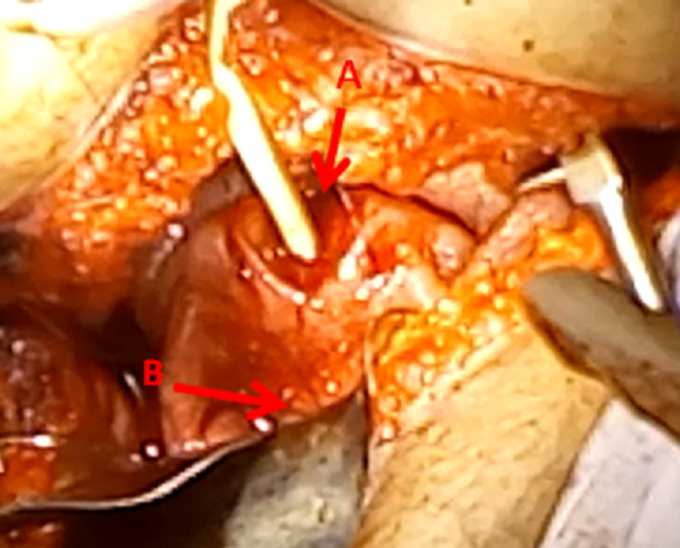
surgical view: (A) creation of a utero vaginal canal; (B) anastomosis site

A 17-year-old virgin girl referred to our tertiary center with a story of primary amenorrhea and severe cyclic abdominal pain occuring over a period of three years. General examination results were normal and secondary sexual characteristics were well developed. The hymen was intact and had normal perforations. Ultrasound was suggestive of hematometra with right endometrioma of 7 x 7cm. Laparoscopy was proposed and showed a low abundance of brownish blood suggesting tubal reflux, the uterus and appendix were normal. A cystectomy was done. Magnetic resonance imaging (MRI) showed a normal endometrial cavity and the absence of cervical canal; the vagina was normal (Figure 1). Informed consent from the patient's parents was obtained after verbal counseling and an explanation of the potential risks of the surgery. A laparotomy was performed under general anesthesis. Intra-operatively, uterus was enlarged with normal bilatéral fallopian tubes, ovaries were normal. The proximal end of vagina was exposed after dissection of the visceral peritneum between uterus and the bladder. Midline incision in the isthmus was done to reach the caudal portion of endometrial cavity. A utero-vaginal anastomosis was successfully performed (Figure 2). The patient´s postoperative course was without problems. Menses were observed the first month after the operation. Subsequently, our patient continued to have regular periods while we followed her for the next six months. The pelvic ultrasound examination was normal.

## Discussion

The incidence of cervical agenesis is 0.01% in general population [1]. It represents about 3% of all uterine anomalies [2]. It is classified as type IB in the American fertility society classification of mullerian anomalies [3]. According to the new European Society of Human Reproduction and Embryology (ESHRE) and European Society for Gynaecological Endoscopy (ESGE) classification systems cervical agenesis is classified as U_0_C_4_V_0_ [4]. It is rarely associated with the presence of normal vagina and functioning uterus and if associated with functioning uterus, hematometra will occur. Approximately, 4.8% of women with cervical agenesis have a functioning uterus [5]. Various imaging tools have been used to assess anomalies of the Mullerian canals. Currently, magnetic resonance imaging (MRI) is the best choice for the definitive diagnosis and classification of these anomalies [6]. The conservative surgical approach of these patients involves a uterovaginal anastomosis. In fact, these patients generally preserve their fertility and need a procedure that supports cyclical rules and does not allow restenosis [7]. Unfortunately, hysterectomy might be necessary when the conservative treatment fails [8]. Cervicoplasty with mucosal lining permits the creation of a patent cervical canal, even in the reputedly unfavorable forms of congenital cervical agenesis [9].

## Conclusion

Cervical agenesis is an anomaly that must be considered in front of any primary amenorrhea despite its rarity and this to start an adequate management because the impact on the reproductive potential of a woman can be significant.
